# Right-to-left shunts and hormonal therapy influence cerebral vasomotor reactivity in patients with migraine with aura

**DOI:** 10.1371/journal.pone.0220637

**Published:** 2019-08-01

**Authors:** Claudia Altamura, Matteo Paolucci, Nicoletta Brunelli, Angelo Cascio Rizzo, Gianluca Cecchi, Federica Assenza, Mauro Silvestrini, Fabrizio Vernieri

**Affiliations:** 1 Clinical Neurology, Headache and Neurosonology Unit, Campus Bio-Medico University of Rome, Roma, Italy; 2 Neurological Clinic, Department of Experimental and Clinical Medicine, Marche Polytechnic University, Ancona, Italy; University of L’Aquila, ITALY

## Abstract

Patent Foramen Ovale and impaired cerebral hemodynamics were proposed among the pathophysiological mechanisms explaining the increased risk for stroke in patients with Migraine with Aura (MA). Our study aimed at comparing the vasomotor reactivity (VMR) of the anterior and the posterior cerebral circulation in patients with Migraine with Aura, in patients with acute vascular ischemic accidents, and in controls. We hypothesized that VMR in MA patients is preserved in the anterior circulation and reduced in the posterior circulation. We prospectively assessed with Transcranial Doppler the vasomotor reactivity to breath holding of the Middle and Posterior Cerebral Arteries (MCA, PCA) in MA patients, in acute vascular patients and healthy controls. We also evaluated the possible effect of clinical characteristics of MA (attack frequency, aura length or type, disease history), vascular factors and the presence of right-to-left shunt on VMR. Diverging from our hypothesis, MA patients displayed a higher breath-holding index (BHI) than controls in the MCA (1.84±0.47%/s vs 1.53±0.47%/s, p = .001) as well as in the PCA (1.87±0.65%/s vs 1.47±0.44%/s, p < .001). In MA patients, MCA BHI was higher in those with large right-to-left shunts (2.09±0.42 vs 1.79±0.47, p = .046) and lower in those taking estrogens (1.30±0.30%/s vs 1.9±0.45%/s, p = .009). We did not observe an effect of MA characteristics on BHI. The increased BHI in MA patients with large right-to-left shunts could be explained by the vasoactive effect in the cerebral circulation of substances bypassing the deactivating pulmonary filters or by a constitutional trait of the vascular system associating persistent right-to-left shunts and hyper-reactive hemodynamics. Our results discourage the hypothesis that altered hemodynamics contribute to increasing the stroke risk in all MA patients. However, estrogens can lower VMR, curtailing the hemodynamic resources of MA patients.

## Introduction

Migraine and more specifically Migraine with Aura (MA) is associated with an increased risk of ischemic stroke, [1] that increases exponentially in smokers and in patients taking oral contraceptives.[2] Among other mechanisms, high prevalence of patent foramen ovale (PFO) and of genetic thrombophilic mutations and impairment of cerebral hemodynamics have been proposed to subtend this pathological link.[3]

Vasomotor Reactivity (VMR) is a marker of the efficiency and health of cerebral circulation: it reflects the potential of the intracranial arterioles to dilate in response to vasodilatory stimuli, such as hypercapnia. VMR impairment has been associated with increased stroke risk in patients with carotid steno-occlusive diseases [4]. Studies addressing cerebral hemodynamics in MA patients produced contradictory results:[5,6] some reported that VMR is preserved or even increased in the anterior circulation [7–9] while that is less efficient posteriorly.[10–12] Consistently, migraine patients may display an increased subclinical vascular burden at magnetic resonance imaging mainly in the posterior territory.[13] Nevertheless, recent reports refuted this finding, also in patients with right-to-left shunts (RLS)[14] or with migraine with aura [15]. Right-to-left shunts. i.e the mixture of the arterial and venous blood, derive from lung arterio-venous malformations or from cardiac origins. PFO is the most frequent locus of right-to-left shunt of cardiac origin, is present in around 25–30% of the adult population and is usually clinically silent.[16]

While previous studies addressed the possible influence of RLS and clinical characteristics of MA on cerebral autoregulation [6,17], the possible modifiers of VMR in MA patients have not been explored before. Since an efficient VMR may protect MA patients from the risk of stroke, the identification of modifiable factors influencing VMR would have a relevant clinical significance.

Our study aimed at comparing the cerebral hemodynamics of the anterior and the posterior circulation in patients with MA, patients with acute stroke/motor TIA, and controls. We also investigated whether RLS and other clinical factors are associated with an impairment of VMR in MA patients. Based on a critical revision of the results of previous studies about the issue, we hypothesized that VMR to hypercapnia in MA patients was comparable to healthy controls in the anterior circulation, and to stroke patients in the posterior circulation.

## Material and methods

This observational study was carried out at Headache and Neurosonology Unit of the Neurological Clinic at the Campus Bio-Medico University of Rome.

### Standard protocol approvals, registrations, and patient consents

The study was conducted according to the ethical guidelines of the Helsinki Declaration of 1975 (and as revised in 1983) and was approved by the Ethics Committee of the Campus Bio-Medico University of Rome (Prot. 53/18). All participants provided their written informed consent.

### Study protocol

We consecutively enrolled MA patients, young (younger than 60 years) patients with cryptogenic minor stroke or motor TIA among subjects referred to our Neurosonology lab to undergo Transcranial Doppler (TCD) bubble test to detect RLS. All vascular (stroke/TIA) patients were referred to our Neurosonology lab from our Neurology ward where they were admitted for the sudden onset of motor deficits. Since migraineurs experiencing cerebral ischemia were reported to be affected more frequently by cryptogenic stroke,[18] patients with cryptogenic minor stroke/TIA (NIHSS≤3) [19]were enrolled in this study to compare MA patients with subjects with a possible mild impairment of cerebral hemodynamics. We also enrolled healthy controls sex and age-matched with MA patients among subjects undergoing the TCD bubble test for a suspected PFO at screening echocardiography or for professional reasons (e.g professional divers and athletes) among personnel volunteering to perform BH test. We considered exclusion criteria for controls: any present or previous neurological deficit and cardiological symptoms, history of Migraine, radiological evidence of leukoencephalopathy.

Migraine with Aura was diagnosed according to The International Classification of Headache Disorders.[20] Extra- and intra-cranial arteries were assessed by continuous wave Doppler and color flow B-mode Doppler ultrasound (Philips iU22, Bothell, WA, USA). Subjects with extra- and intra-cranial arteries stenosis were excluded from the study. TCD examinations for RLS detection were performed according to guidelines (Multidop-X DWL; ElektronischeSysteme GmbH, Germany).[21]

We defined RLS grade as follows: small-medium size:< 20 hyperintense transient signals (HITS) at rest or “shower” effect after Valsalva Maneuver; large size: “shower” at rest and/or “curtain” effect after Valsalva Maneuver or “curtain” effect at rest.

To define RLS of presumed cardiac origin we considered only HITS observed within 30 seconds from microbubbles injections, as delayed HITS are suggestive of arterio-venous lung shunts.

Of the 98 subjects undergoing TCD testing for RLS detection, 69 had also received transthoracic (without microbubble injection) or transesophageal echocardiography.

Cerebral vasomotor reactivity (VMR) to hypercapnia was measured using the breath-holding index (BHI).[22] VMR evaluation preceded TCD microbubble test in all subjects. BH test was performed within 7 days and at least 48 hours from symptom onset in vascular patients and always in the inter-ictal period in MA patients. In stroke patients, PCA and MCA were insonated in the unaffected territory/side, while in MA patients and controls, PCA was insonated at the right side and MCA on the left.

All examinations were carried out in a supine position. All subjects were instructed for a night fasting (including alcohol drinking) and abstinence from nicotine-smoking or drug use for at least 12 hours. Two transducers placed on the temporal bone window, hold on a headband, with a stable angle of insonation were used to obtain a bilateral continuous measurement of the flow velocity of middle and posterior cerebral arteries. The highest flow signal was sought at a depth of insonation ranging from 48 to 54 mm for the middle cerebral artery (MCA) and from 55 to 70 mm for the posterior cerebral artery (PCA). Subjects were requested to hold their breath for a period of 30 s which was monitored by a capnometer.

BHI was calculated by dividing the percent increase in mean flow velocity (MFV) occurring during breath-holding by the length of time (in seconds) that subjects held their breath after a normal inspiration ([MFV at the end of breath-holding -rest MFV/rest MFV] X100/seconds of breath-holding). Traces from subjects who could not hold his breath for at least 30 s were discarded. A neuro-sonologist blind to subjects’ diagnosis computed BHI off-line. We used, as pathological BHI, a cutoff value less than 0.69%/s because it has clearly been demonstrated to have a high predictive value for cerebrovascular disease.[23]

We recorded vascular risk factors, including current estrogen intake and smoking, in all subjects and, when available, the results of Factor II, Factor V, and MTHFR genotyping, lupus anticoagulant antibodies, homocysteine blood levels, and echocardiography for atrial septal aneurysm (ASA) detection. Hypertension and diabetes were defined according to WHO guidelines (www.who.int). We considered medical history positive for inherited or acquired thrombophilia in case of finding of: homozygosis or heterozygosis for Factor II and/or Factor V and/or homozygosis/double heterozygosis (C677T-A1298C) for MTHFR and/or homocysteinemia above the upper reference range of our laboratory (15 umol/L) and/or detection of lupus anticoagulant antibodies.

### Statistical analysis

This is the primary a priori analysis of these data. No statistical power calculation was conducted prior to the study, as the sample size was based on our previous experience with this design and further amplified.[7,24] Subjects’ characteristics, such as age, MFV, BHI, homocysteinemia, were stored as interval variables. Sex, smoking, diabetes, hypertension, estrogen intake, inherited thrombophilia and ASA were collected as binary variables.

The analyses were performed for interval variables with independent t-test or Mann–Whitney test for comparisons between groups and with Spearman or Pearson tests for correlations according to the results of the Kolmogorov-Smirnov test for data distribution. Measures of central tendency (e.g., mean, median) and variability (SD, IQR) were used to report interval variables according to respectively to normal or not-normal distribution. Binary variables were analyzed with the Chi-squared test. Statistical significance was set at two-tailed p<0.05. ANOVA and Multiple logistic regression analyses were used to assess possible influencer of BHI. A generalized linear model was run to assess the possible interaction between the presence of moderate-severe RLS and patients’ age or disease history in influencing BHI.

Statistical analyses were performed with SPSS 25.0; SPSS Inc., Chicago, IL, USA.

## Results

56 MA patients, 20 patients with cryptogenic minor stroke (n = 12) or motor TIA (n = 8), and 53 healthy controls sex and age-matched with MA patients (22 undergoing the TCD bubble test and VMR assessment and 31 only VMR evaluation).

Age, MCA and PCA MFVs, as well as MCA BHI, resulted distributed normally. All the other interval variables did not present a normal distribution.

The basal MCA MFV was highly correlated to the basal PCA MFV in controls (Pearson correlation = .556, p<0001), in MA subjects (Pearson correlation = .320, p = .016) and vascular patients (Pearson correlation = .625, p = .003). Similarly, MCA BHI was strongly associated with PCA BHI in all groups (ρ = .748, p < .0001 in controls; ρ = .680, p < .0001 in MA patients; and ρ = .531, p = .016 in vascular patients).

### Comparison across groups

Table 1 summarizes patient clinical profiles and the results of the BH test. MA patients did not differ from controls for age and sex. Patients with stroke/TIA were significantly older than MA patients (p = .002) and controls (p = .001) and were more frequently male (p = .003).

**Table 1:** Patient clinical profiles.

	CONTROLS	MA	YOUNG STROKE/TIA
**age (years, mean and SD)**	35.3 (13)	36.6 (10)	46.0 (10)*
**sex (F)**	79.2%	82.1%	45.0%*
**smoking habit**	15.2%	10.7%	22.2%
**diabetes**	0%	0%	0%
**hypertension**	17.0%	13.2%	20.0%
**estroprogestinic therapy (%F)**	20.0%	16.7%	12.5%
**thrombophilia**	30%	35%	43%
**homocysteine (mmol/L, median and IQr)**	19.1 (7.1)	10.0 (4.7)	10.3 (10.0)
**RLS (%)**	33.3%	59.1%*	95.0%**
**severe RLS (%)**	9.5%	23.6%	25.0%
**atrial septal aneurysm (%)**	45.4%	27.0%	66.6%*
**MCA basal MFV (cm/s, mean and SD)**	70.9 (16.6)	68.4 (12)	60.2 (10)*
**PCA basal MFV (cm/s, mean and SD)**	48.0 (13.8)	46.8 (12)	41.7 (12)
**BHI MCA (%/s, mean and SD)**	1.53 (0.47)	1.84 (0.47)*	1.47 (0.63)
**BHI PCA (%/s, mean and SD)**	1.47 (0.44)	1.87 (0.65)*	1.37 (0.47)

BHI = breath-holding index, F = female, MA = migraine with aura, MCA = Middle Cerebral Artery, MVF = mean flow velocity, PCA = Posterior Cerebral Artery, RLS = right-to-left shunt, SD = standard deviation, IQr = interquartile interval.

*p < .05,

**p < .001.

Groups did not differ in the prevalence of the vascular risk factors, thrombophilic conditions and for homocysteinemia.

Vascular patients presented a higher prevalence of RLS (p < .001) and ASA (p = .041) compared with MA patients and controls. The presence of large RLS was equally distributed across groups. Vascular patients presented lower basal MCA MFV than MA patients (p = .009) and controls (p = .002), while no difference was observed for the basal PCA MFV.

The results of microbubble TCD agreed with the available echocardiographic findings for the presence of patent foramen ovale (p = .018)

VMR was preserved in all groups as average BHI was largely above the pathological cutoffs in the MCA (0.69%/s) as well as in the PCA.[23]

BHI in the MCA and in the PCA was significantly higher in MA patients than in controls (MCA BHI, p = .001; PCA BHI p<001) and in vascular patients (MCA BHI, p = .007; PCA BHI p = .001). The ANOVA including age (as covariate) and sex and diagnosis (as fixed factors) confirmed this finding (ANOVA: MCA BHI: F (2,122) = 5.700, p = 0.004; PCA BHI: F (2,122) = 6.884, p = .001) also after Bonferroni post hoc testing (consistently p < .005 for the comparison of MCA and PCA BHIs in migraine patients with stroke and controls).

**Fig 1:**
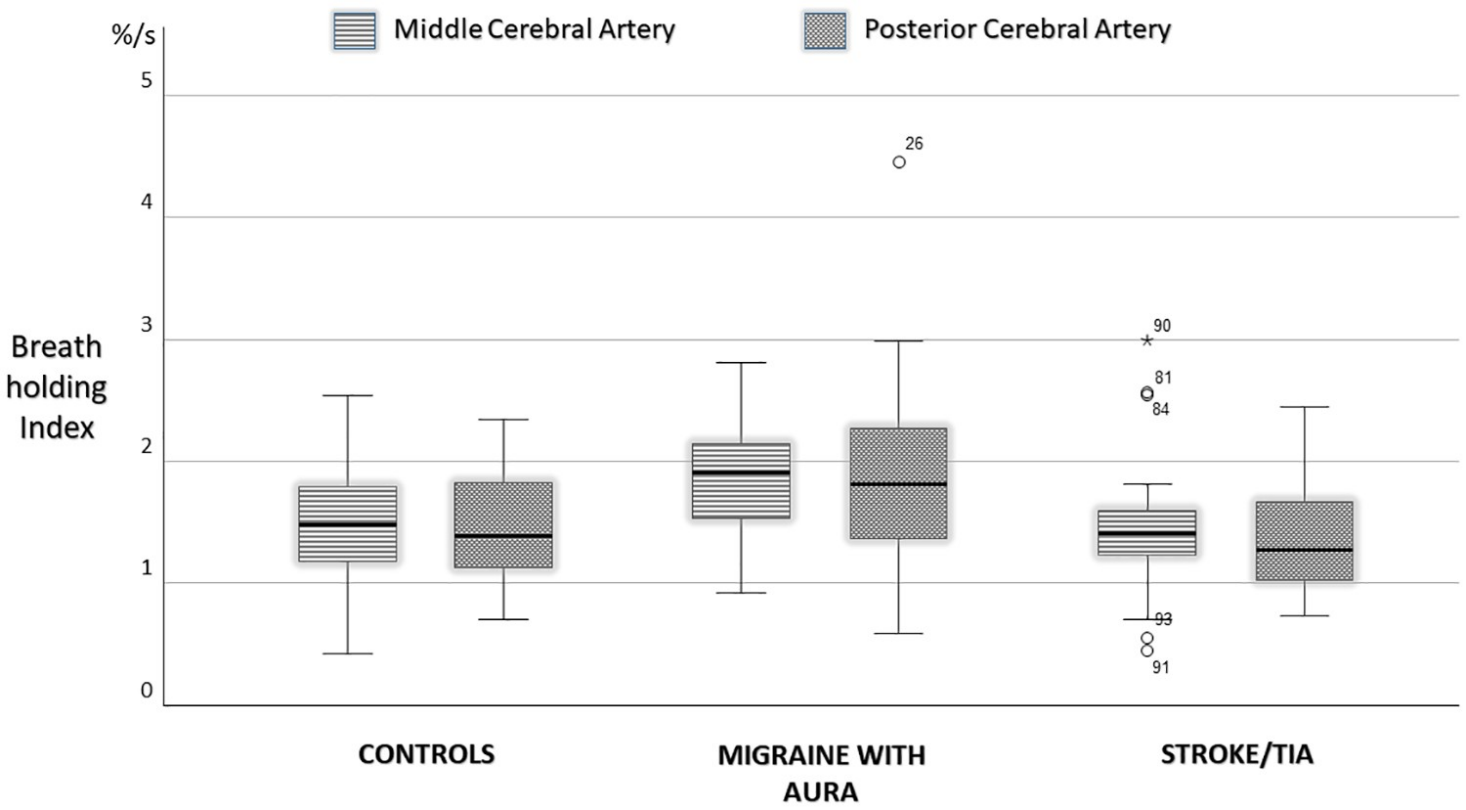
Breath holding index in the middle and posterior cerebral arteries compared in controls, patients with migraine with aura and vascular patients.

MCA and PCA BHI were similar in controls and vascular patients (Fig 1).

Since RLS was not investigated in all controls, we did not address its possible association with BHI variability in this group. However, mean MCA and PCA BHI indices in the controls undergoing also RLS investigation did not differed from those undertaking only BH test.

### VMR association with clinical factors in MA patients

Visual aura was reported by 55.4% of patients and Aura with multiple symptoms in the remaining cases. MA attacks occurred with a mean frequency of 12.4 per year (SD 20.7); the mean length of aura phenomenon was 36.5 min (SD 27.3). Disease history was 15.7 years (SD 13.7).

In MA patients, the results of microbubble TCD agreed with the available echocardiographic findings for the presence of patent foramen ovale (p = .006). The divergent findings were due to 15 out of 39 examinations where RLS did not correspond to the diagnosis of PFO at transthoracic echocardiography without microbubble injections.

RLS were confirmed as PFO at transesophageal examination in 100% of cases.

**Fig 2:**
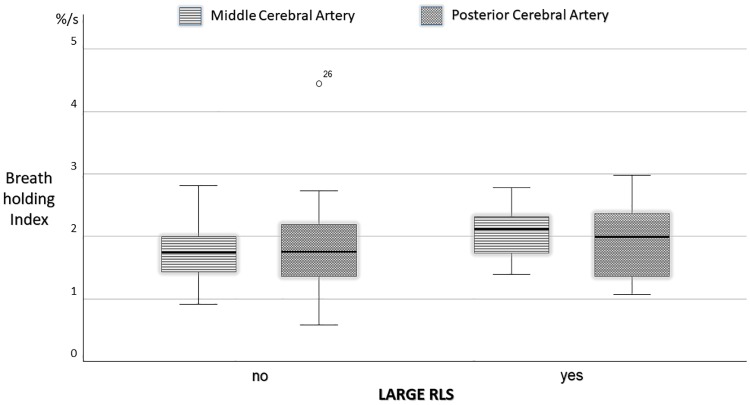
Breath holding index in the middle and posterior cerebral arteries in patients with migraine with aura compared for the presence of large right-to-left shunts.

In MA patients, MCA and PCA BHI did not differ when compared by type of aura. In MA patients, large RLS were associated with higher BHI in the MCA (2.09±0.42%/s vs 1.79±0.47%/s, p = .046, Fig 2).

**Fig 3:**
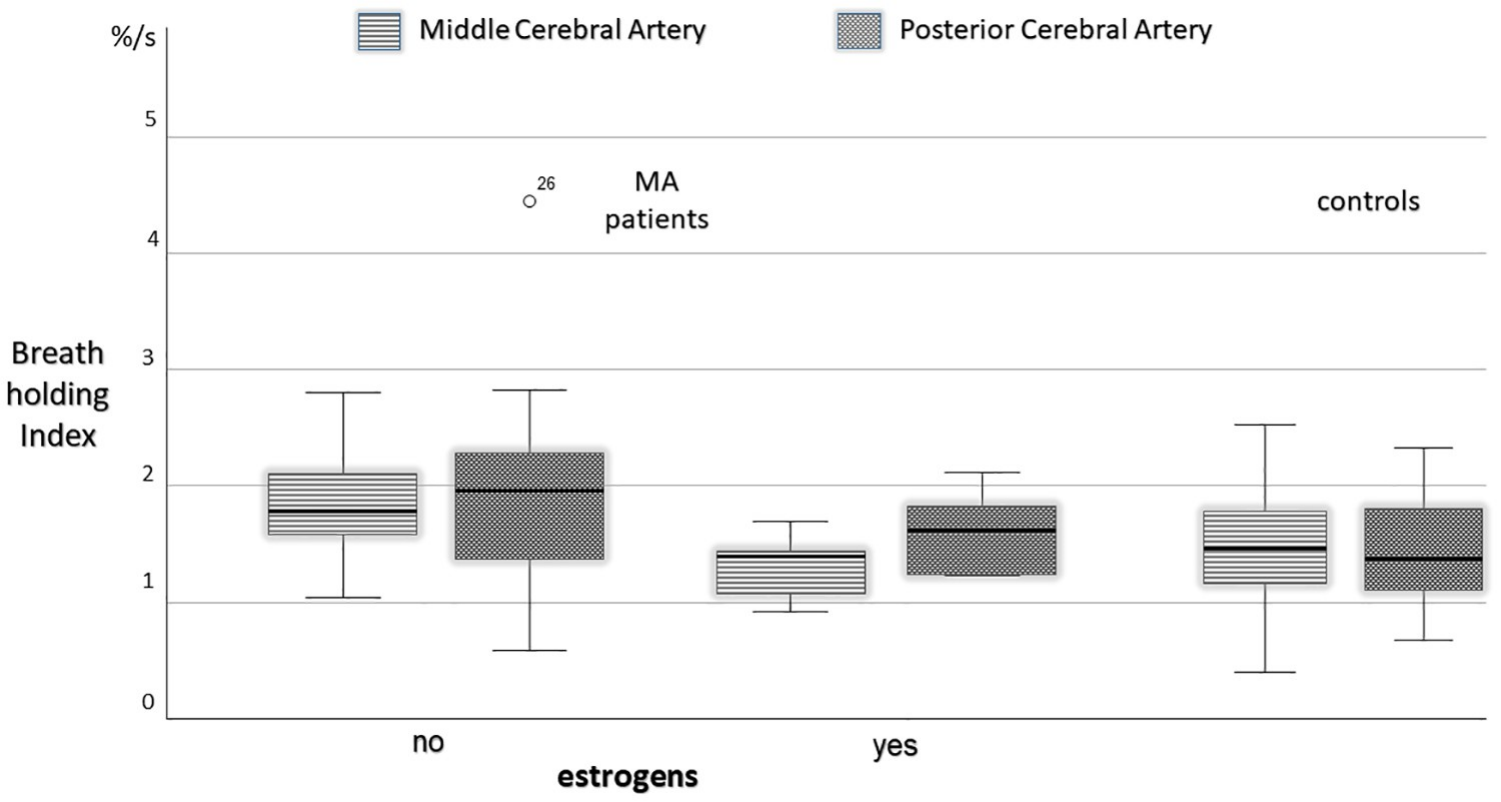
Breath holding index in the middle and posterior cerebral arteries in patients with migraine with aura compared for estrogen intake, and in healthy controls.

Besides, MA patients on estrogen therapy showed lower BHI in the MCA than those who did not (1.30±0.30 vs 1.9±.045%/s, p = .009, Fig 3). Also controls on estrogen therapy displayed a lower MCA BHI (1.25±0.53 vs 1.53±0.44%/s,) but this finding was not significant. Although no statistically significant, mean MCA BHI in MA patients on estrogen therapy was lower than MCA BHI in controls (Fig 3). Multiple regression analysis confirmed the effect of estrogen therapy on MCA BHI (dependent variable), while MA clinical characteristics (attack frequency, duration of aura phenomenon, disease history) and smoking or hypertension did not influence MCA BHI (Table 2). To note, estrogen therapy was also associated with a higher basal MCA (78.8±14.5 vs 67.3±11.5 cm/s, p = .042) in MA patients and with a non significant trend in controls (81.6±10.9 vs 68.9±17.5 cm/s).

**Table 2:** Simple and Multiple linear regression of MCA BHI on Migraine with Aura clinical characteristics, risk factors, and right-to-left shunt.

	Simple linear regression					Multiple linear regression						
	B	SE	Beta	t	Sign	B	SE	Beta	t	Sign	95% CI	
											lower limit	upper limit
disease history (years)	.002	.005	.060	.433	.667	.002	.005	.053	.349	.729	-.009	.013
MA attack frequency (n/years)	.001	.003	.056	.403	.688	.000	.003	-.009	-.062	.951	-.006	.006
aura duration (min)	-.003	.002	-.144	-1.056	.296	-.004	.002	-.218	-1.567	.125	-.009	.001
smoking habit	.036	.206	.024	.174	.863	.039	.227	.026	.174	.863	-.418	.497
estroprogestinic therapy	**-.599**	**.208**	**-.365**	**-2.883**	**.006**	**-.547**	**.220**	**-.359**	**-2.482**	.017	-.992	-.102
hypertension	-.099	.214	-.064	-.461	.647	-.221	.227	-.145	-.974	.336	-.680	.238
severe RLS	**.304**	**.145**	**.277**	**2.096**	**.041**	.200	.145	.190	1.377	.176	-.093	.492

## Discussion

Although the hypothesis that the migraine generator lies in an impaired hypothalamus-brainstem connectivity has largely achieved consensus [25], the vascular involvement in migraine cannot be neglected. The different phases of migraine are characterized by changes in brain activity in the somatosensory cortex, brainstem, and thalamus, as well as by cerebral blood flow perturbations. [26,27] Moreover, the perivascular neurotransmitters calcitonin gene-related peptide-CGRP and pituitary adenylate cyclase-activating peptide-PACAP, that regulate the cerebral blood flow with a vasodilatory effect, have been largely involved in migraine pathophysiology[28] and are the target of brand-new approved preventive treatments and of drugs currently in development.[29]

Disordered hemodynamics and paradoxical embolism have been proposed among other mechanisms to explain the increased risk of stroke in migraine patients, principally in patients with MA.[3,30] Besides, when migraine patients, particularly those with MA, experience stroke they display a reduced mismatch between the infarcted and the hypoperfused territories, suggesting a high vulnerability to the ischemic damage.[31,32] In this scenario, it would be important to establish the hemodynamic profile of MA patients.

In this study, we evaluated cerebral VMR to hypercapnia (BH test) in the MCA and in the PCA in a large group of MA patients in the interictal phase. Based on previous studies [5,8–10,33], we had hypothesized that VMR was preserved in the anterior circulation and reduced in the posterior circulation. Differently from our starting hypothesis, we observed that MA patients present a higher BHI in the anterior but also in the posterior circulation compared with controls.

Cerebral hemodynamics is the peculiar property of the brain circulation that allows to guarantee a constant tissue perfusion in different situations. Brain arterioles can regulate their caliper in response to the endothelial paracrine secretion, to variations in systemic blood pressure (i.e. cerebral autoregulation) and in CO_2_/O_2_ concertation (i.e. VMR), and to neural activation. [34] To note, while cerebral extra-parenchymal vasculature receives a dense sensitive, sympathetic and parasympathetic neuronal innervation, parenchymal vessels supply adequate blood inflow in response to neural metabolic demands mainly mediated by astrocyte production on nitric oxide (NO) and prostaglandins (i.e. neurovascular coupling).[28,35]

In the presence of a biochemical trigger (i.e. CO2), MA patients in the inter-ictal phase present more prominent arteriole vasodilation than controls. This may due to an increased release of vasoactive substances (e.g. NO) by the endothelium or to an increased sensitivity of vessel smooth muscles as theorized by Olesen.[36,37] One possible explanation for these findings is an imbalance between parasympathetic (donor of NO and other vasodilating agents) and sympathetic activity.[38] Interestingly, we have previously observed a more reactive vascular tree, measured with flow-mediated dilation, also in the peripheral circulation suggesting it is a constitutional characteristic of MA patient vessels.[33]

In addition, we observed for the first time that MA patients with clinically relevant RLS display a higher BHI in the MCA than MA patients without or less severe RLS. A non-significant difference was observed in the PCA (Fig 2).

The effect of RLS on cerebral hemodynamics was observed by a previous study describing an impaired spontaneous dynamic autoregulation in migraineurs with large RLS.[17] This result apparently contrasts our findings, but indeed it may be interpreted as an excessive compliance of cerebral vessels to the dynamic variations of blood pressure that can somehow resemble the higher sensitivity to CO2 we have observed in the present study.

The increased BHI in MA patients with large right-to-left shunts could be mediated by the vasoactive effect in the cerebral circulation of substances (e.g. serotonin, CO2) bypassing the deactivating pulmonary filters, or by a constitutional trait of the vascular system associating persistent RLS and hyper-reactive hemodynamics.

Alternatively, we may speculate that the higher BHI may represent the effect of ischemic preconditioning to the frequent transit of microemboli in the cerebral circulation. Interestingly, Gollion and colleagues recently reported a better cerebral autoregulation efficiency in MA patients with longer disease history.[6] We fail to find any association between MA clinical characteristics and vasomotor reactivity, suggesting that the higher BHI observed in MA is a constitutional trait.

Nevertheless, MA patients on estrogen therapy presented a less valid vasomotor-reactivity compared to the other MA subjects. Physiological oscillations of estrogen have been described to modify VMR in healthy women, which display a higher VMR in the ovulatory period in correspondence to the estrogenic peak production[39]. This observation is consistent with several experimental studies demonstrating that estrogens exert a vasodilatory effect on the vascular bed mediated by different mechanisms including endothelial production of NO, prostaglandins and serotoninergic modulation[40]. These mechanisms explain the well-known protective vascular effect of endogenous estrogens. As a paradox, exogenous administration of estrogens increase the stroke risk in reproductive ages as well as in menopause [41]. Exogenous estrogens seem to be even more hazardous in MA patients [2,42,43] Our finding helps to clarify why, among other vascular factors, estrogens represent a major risk factor for stroke in women with MA: beyond the pro-thrombotic hazard, estrogens may have a detrimental effect on cerebral hemodynamics curtailing the defensive resources of MA patients. A possible explanation of this finding is that exogenous estrogens are less effective than the endogenous estrogens, whose production is suppressed, in refining the regulation of vascular tone. To note, in our cohort of MA patients, basal MCA flow velocity was higher in those on hormonal therapy. This may reflect a persistent condition of arteriolar vasodilation that would limit the capability of vessels to further dilate if needed.

This study has some limitations. Since TCD for RLS detection implies the venous injections of microbubbles, to ensure a correct comparison for age and sex, controls were enrolled among subjects undergoing the microbubble test for a clinical indication and among staff personnel volunteering to undergo only BH test.

Despite the statistical significance, the relatively small number of MA patients wth RLS and/or esntrogenic therapy requires confirmation of our observations in a larger population. Finally, MA patients on hormonal therapy took different estrogenic formulations. A specific analysis of the effect of different formulations was not possible given the limited number of patients. However, for ethical reasons, it is not possible to design an interventional trial on this topic because of the vascular risks connected with estrogens in MA patients. In fact, the enrolled patients were instructed to discontinue hormonal therapy.

Overall, the results from the present study underline a crucial point in the complex relationship between MA and stroke: if the cerebral hemodynamics plays a role it is protective, at least outside the migraine attack.

## Supporting information

S1 Dataset Complete dataset. (SAV)

S1 Strobe Checklist Strobe checklist. (DOC)
